# The Effect of Preoperative Benzodiazepine Usage on Postoperative Opioid Consumption After Hand Surgery: A Multicenter Analysis

**DOI:** 10.7759/cureus.29609

**Published:** 2022-09-26

**Authors:** Brock K Bakewell, Clay B Townsend, Justin A Ly, Matthew Sherman, Hasham M Abdelfattah, Mark Solarz, Katharine Woozley, Asif M Ilyas

**Affiliations:** 1 Foundation for Opioid Research and Education, Rothman Orthopedic Institute, Philadelphia, USA; 2 Foundation for Opioid Research and Education, Rothman Orthopaedic Institute, Philadelphia, USA; 3 Orthopedic Surgery and Sports Medicine, Temple University Hospital, Philadelphia, USA; 4 Orthopaedic Surgery and Sports Medicine, Temple University Hospital, Philadelphia, USA; 5 Department of Orthopaedics, Einstein Healthcare Network, Philadelphia, USA; 6 Division of Hand Surgery, Rothman Orthopedic Institute at Thomas Jefferson University, Philadelphia, USA

**Keywords:** pain management, thumb basal joint arthroplasties, distal radius fracture open reduction internal fixations, carpal tunnel releases, benzodiazepines, opioid

## Abstract

Background

Prescription rates of opioids and benzodiazepines have steadily increased in the last decade with the percentage of prescription opioid overdose deaths involving benzodiazepines more than doubling during that time. Orthopaedic surgery is one of the highest-volume opioid prescribing medical specialties, but the effects of benzodiazepine use on orthopaedic surgery patient outcomes are not well understood. The purpose of the study was to utilize the state Prescription Drug Monitoring Program (PDMP) database to investigate if perioperative benzodiazepine use predisposes patients to prolonged opioid use following hand and upper extremity orthopaedic surgery.

Methods

This study was retrospective and conducted at three urban academic institutions. All patients who underwent carpal tunnel release, thumb basal joint arthroplasty, and distal radius fracture open reduction internal fixation performed by 14 board-certified, fellowship-trained orthopaedic hand and upper extremity surgeons between April 2018 and August 2019, were collected via a database query. All opioid and benzodiazepine prescriptions were collected from three months preoperatively to six months postoperatively.

Results

In this study, 634 patients met the inclusion criteria presented to one of the three institutions during the 18-month study period. Patients consisted of 276 carpal tunnel releases, 217 distal radius fracture open reduction internal fixations, and 141 thumb basal joint arthroplasties. Benzodiazepine users were 14.6% more likely to fill an additional opioid prescription (p<0.005) and were 10.8% more likely to experience prolonged three to six-month postoperative opioid use (p<0.005).

Conclusion

This study found that patients who use benzodiazepines are at a higher risk of filling additional opioid prescriptions and prolonged opioid use following hand and upper extremity surgery. Prescribers should take this into account when prescribing opioids after upper extremity orthopaedic surgery.

## Introduction

Since the start of the opioid epidemic two decades ago, hundreds of thousands of Americans have died from opioid-related drug overdose, with opioids being involved in almost 50,000 overdose deaths in 2019 alone [1]. Despite this, opioid prescribing has increased by 350% over the last two decades [1-3]. Over the same time, the prescription rates of benzodiazepines have also increased substantially. From 1996 to 2013, the number of American adults filling benzodiazepine prescriptions increased by 67% to an estimated 13.5 million adults annually [4]. Studies have shown that when opioids and benzodiazepines are used concurrently, they enhance each other’s pharmacologic effects, increasing the risk of fatal overdose [5,6]. From 2000 to 2017, with prescription rates of both opioids and benzodiazepines increasing, the percentage of prescription opioid overdose deaths that involved benzodiazepines more than doubled from 15.6% to 33.1% [5].

Orthopaedic surgery is one of the highest-volume opioid prescribing medical specialties, following family medicine and internal medicine [7]. Although opioids are often helpful in acute postoperative pain control, orthopaedic surgery patients remain at risk for developing long-term opioid dependence and other adverse effects related to opioid use [8]. It is well established in the current literature that orthopaedic surgery patients who use opioids preoperatively are at higher risk for prolonged opioid use, poorer surgical and functional outcomes, and increased risk for complications [9-12]. However, the effects of benzodiazepine use on orthopaedic surgery patient outcomes remain seldom studied and poorly defined.

State-run Prescription Drug Monitoring Program (PDMP) database websites provide physicians with access to their patients' extensive controlled substance prescription history [13]. This invaluable resource has certainly prevented patients from receiving excessive controlled substances from multiple medical providers and has helped surgeons to predict better which patients are at increased risk for prolonged opioid use postoperatively. The purpose of the current study was to utilize the state PDMP database to investigate if perioperative benzodiazepine use predisposes patients to prolonged opioid use following hand and upper extremity surgery. We hypothesized that patients who used benzodiazepines would have higher rates of opioid prescription refills and prolonged opioid use postoperatively.

## Materials and methods

Approval was received from the Thomas Jefferson University Institutional Review Board prior to beginning this retrospective study. This study was conducted at three urban academic namely institutions namely (1) Rothman Orthopaedic Institute, Thomas Jefferson University, Philadelphia (2) Department of Orthopaedic Surgery and Sports Medicine, Temple University Hospital, Philadelphia, and (3) Department of Orthopaedics, Einstein Healthcare Network, Philadelphia, from 4/30/2018 to 8/30/2019. Information on all the patients who underwent carpal tunnel release (“CTR”, Current Procedural Terminology {CPT} 64721), basal joint arthroplasty (“BJA”, CPT 25447), and distal radius fracture open reduction internal fixation (“DRF ORIF”, CPT 25609) performed by 14 board-certified, fellowship-trained orthopaedic hand, and upper extremity surgeons was collected via a database query. This date range was selected to prevent the study period from overlapping with COVID-19 lockdowns, which may represent inconsistencies in standard prescribing trends. Utilizing the Pennsylvania Prescription Drug Monitoring Program (PDMP) website, all opioid and benzodiazepine prescriptions from three months preoperatively to six months postoperatively were collected. Specific data collected include the type of benzodiazepine prescribed, the type of opioid prescribed, prescription strength, prescription date, the number of pills prescribed, the duration of the prescription, and milligram morphine equivalents (MME) prescribed per day. 

Categorical data were analyzed with Chi-Square testing or Fisher’s Exact test. Continuous data were tested for normality with Shapiro Wilk Test. Parametric data were compared with the student’s t-test, and nonparametric data were compared with the Mann-Whitney U test. Statistical significance was set at p<0.05.

## Results

There were 634 patients who met inclusion criteria that presented to one of the three institutions during the 18-month study period. This consisted of 276 CTRs, 217 DRF ORIFs, and 141 BJAs. Patients included 196 males (30.9%) and 438 females (69.1%) at an average age of 59.4 years (SD 14.7 years) (Table 1). Preoperative opioid use was observed in 28.5% (181/634) of patients. Perioperative benzodiazepine use was observed in 16.4% (104/634) of patients. Preoperative opioid use plus concurrent benzodiazepine use was observed in 6.3% (40/634) of patients. Three out of 10 patients (30.1%) filled an additional opioid prescription within six months postoperatively, and about one out of seven patients (14.0%) developed prolonged three-six-month postoperative opioid use.

**Table 1:** Overall study demographics MME: Milligram morphine equivalents

Variable	n (%) or M (SD)
Age	59.4 (14.7)
Gender	
Male	196 (30.9%)
Female	438 (69.1%)
Initial Postoperative Opioid Prescription Factors	
Total MME/Prescription	139 (137)
Duration in days	4.25 (3.97)
Quantity of Pills	21.6 (14.3)
Preoperative Opioid Use	
No	453 (71.5%)
Yes	181 (28.5%)
Benzodiazepine Use	
No	530 (83.6%)
Yes	104 (16.4%)
Preoperative Opioid Use with Concurrent Benzodiazepine Use	
No	594 (93.7%)
Yes	40 (6.3%)
1+ Opioid Refill Postoperatively	
No	443 (69.9%)
Yes	191 (30.1%)
Prolonged 3-6 Month Opioid Use	
No	545 (86%)
Yes	89 (14%)

When comparing benzodiazepine users versus non-benzodiazepine users, there were no statistical differences in demographics or in the amount and duration of opioids prescribed on the day of surgery (p>0.05) (Table 2).

**Table 2:** Benzodiazepine vs non-benzodiazepine users MME: Milligram morphine equivalents

Variable	Non-Benzodiazepine User N=530	Benzodiazepine User N=104	p value
Age (SD)	59.6 (14.8)	58.5 (14.2)	0.494
Gender			0.261
Female	371 (70.0%)	67 (64.4%)	
Male	159 (30.0%)	37 (35.6%)	
Initial Postoperative Opioid Prescription Factors			
Total MME/Prescription (SD)	138.3 (123.7)	142.7 (190.7)	0.762
Duration in days (SD)	4.2 (3.8)	4.3 (4.8)	0.863
Quantity of Pills (SD)	21.6 (13.6)	21.3 (17.8)	0.821
Preoperative Opioid Use			0.014
No	389 (73.4%)	64 (61.5%)	
Yes	141 (26.6%)	40 (38.5%)	
1+ Opioid Refill			0.003
No	383 (72.3%)	60 (57.7%)	
Yes	147 (27.7%)	44 (42.3%)	
# of Postoperative Opioid Refills	2.3 (2.2)	3.6 (3.9)	0.009
Prolonged 3-6 Month Opioid Use			0.004
No	465 (87.7%)	80 (76.9%)	
Yes	65 (12.3%)	24 (23.1%)	

**Figure 1:**
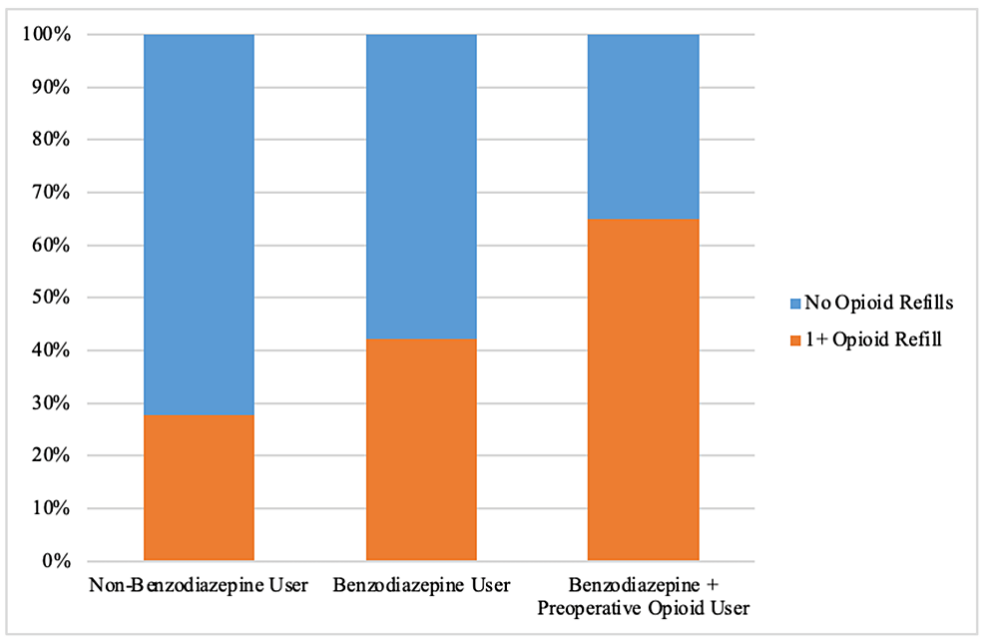
Percentage of non-benzodiazepine users vs benzodiazepine users vs benzodiazepine + preoperative opioid users who filled a second opioid prescription postoperatively.

**Figure 2:**
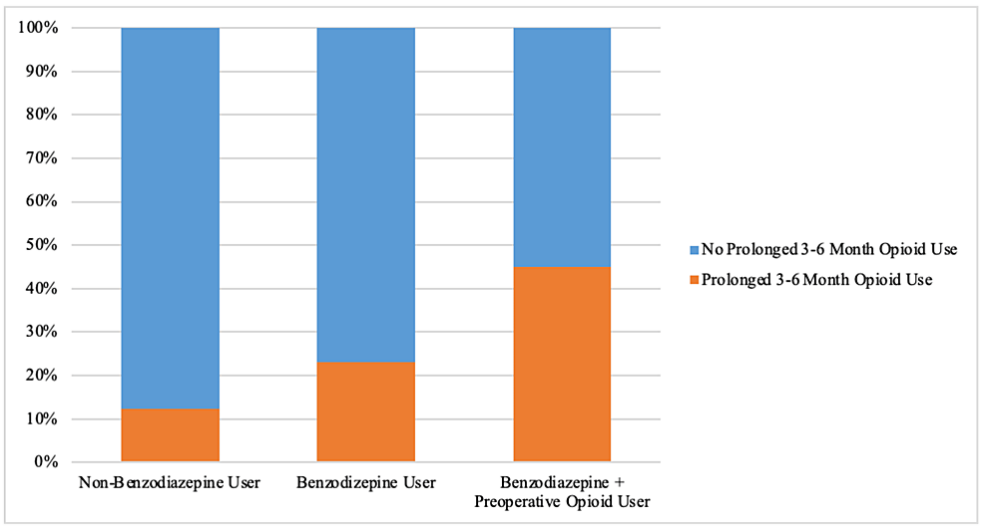
Percentages of non-benzodiazepine users vs benzodiazepine users vs benzodiazepine + preoperative opioid users who developed prolonged 3-6 month postoperative opioid use.

Benzodiazepine users were significantly more likely to fill an additional opioid prescription (42.3% vs 27.7%, p=.003) (Figure 1), and were significantly more likely to experience prolonged 3-6-month postoperative opioid use (23.1% vs 12.3%; p=.004) (Figure 2) than non-benzodiazepine users (Table 2).

**Figure 3:**
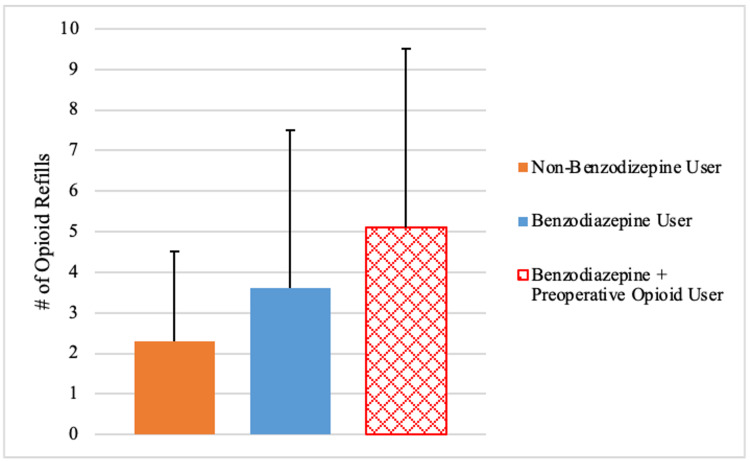
Mean number of opioid refills of non-benzodiazepine users vs benzodiazepine users vs benzodiazepine + preoperative opioid users within 6 months postoperatively, among patients who filled a second prescription.

Additionally, among patients who filled additional opioid prescriptions postoperatively, those who used benzodiazepines filled significantly more opioid prescriptions compared to the non-benzodiazepine users who filled additional opioid prescriptions (3.6 refills vs 2.3 refills; p=.009) (Table 2) (Figure 3).

A sub-analysis was performed of all patients who used opioids preoperatively, comparing those who did or did not use benzodiazepines concurrently. There were no statistical differences in demographics or in the amount and duration of opioids prescribed on the day of surgery between preoperative opioid users that used benzodiazepines versus those that did not use benzodiazepines (p>.05) (Table 3). More opioid plus benzodiazepine users filled additional opioid prescriptions (65.0% vs 47.5%; p=.051) and experienced prolonged three-six-month opioid use (45.0% vs 29.1%; p=.058) compared to opioid users who did not use concurrent benzodiazepines, although these differences were not statistically significant (Table 3) (Figures 1, 2). Of the preoperative opioid users who filled additional opioid prescriptions postoperatively, those who used concurrent benzodiazepines filled significantly more opioid refills compared to those who did not use concurrent benzodiazepines (5.1 refills vs 3.4 refills; p=.032) (Table 3) (Figure 3).

**Table 3:** Preoperative opioid users only vs preoperative opioid users with concurrent benzodiazepine use MME: Milligram morphine equivalents

Variable	Preoperative Opioid User Only (N=141)	Preoperative Opioid Use with Concurrent Benzodiazepine Use (N=40)	p value
Age (SD)	57.9 (15.2)	59.1 (13.4)	0.652
Gender			0.555
Female	99 (70.2%)	30 (75.0%)	
Male	42 (29.8%)	10 (25.0%)	
Initial Postoperative Opioid Prescription Factors			
Total MME/Prescription (SD)	197.0 (198.0)	196.7(284.5)	0.996
Duration in days (SD)	5.5 (6.1)	5.6 (7.2)	0.915
Quantity of Pills (SD)	27.5 (20.5)	26.8 (24.9)	0.862
1+ Opioid Refill			0.051
No	74 (52.5%)	14 (35.0%)	
Yes	67 (47.5%)	26 (65.0%)	
# of Postoperative Opioid Refills	3.4 (2.8)	5.1 (4.4)	0.032
Prolonged 3-6 Month Opioid Use			0.058
No	100 (70.9%)	22 (55.0%)	
Yes	41 (29.1%)	18 (45.0%)	

## Discussion

This study utilized the Pennsylvania Prescription Drug Monitoring Program (PDMP) database to analyze opioid and benzodiazepine prescriptions filled by patients undergoing CTR, BJA, and DRF ORIF across three institutions. Patients who used benzodiazepines perioperatively were found to have significantly higher rates of filling additional opioid prescriptions and developing prolonged opioid use. These findings appeared to be magnified in the preoperative opioid-using patients who also used benzodiazepines. 

The pharmacologic actions of both opioids and benzodiazepines are well established. Opioids act on either Mu, Delta, or Kappa opioid receptors, which are G protein-coupled receptors, and can have a multitude of physiologic effects on the body [14]. Opioid-mediated analgesia occurs mainly via the Mu receptor, and this analgesic response can become blunted in the setting of chronic opioid use, even as soon as after just hours of use [14]. Benzodiazepines are primarily used to treat a variety of psychiatric and neurological conditions, such as anxiety and seizure disorders, via acting on gamma-aminobutyric acid (GABA) receptors to increase chloride flux across cell membranes [15]. 

Current data has indicated that opioids and benzodiazepines are commonly used and abused together [5,6]. Studies have proven that opioids and benzodiazepines modulate each other’s euphoric effects, specifically by increasing the effects of opioids on Mu receptors stimulating the brain’s reward center, which could help explain why these medications are often abused together [5,6,15]. Given the high abuse potential for these medications and the increased risk of fatal overdose when used together, orthopaedic surgeons should be cognizant of these effects and be vigilant when prescribing postoperative opioids. 

Orthopaedic surgeons are among the highest volume opioid prescribers of all medical specialties [7]. While opioids are often necessary for acute analgesia following orthopaedic surgery, prolonged opioid use and addiction can have detrimental effects on both orthopaedic surgical outcomes and long-term health outcomes [16-19]. In a systematic review, Kent et al. evaluated risk factors for persistent postoperative opioid use in patients undergoing total hip arthroplasty (THA) and total knee arthroplasty (TKA) and found that preoperative opioid use was the most prominent risk factor for developing persistent postoperative opioid use [20]. One study identified several risk factors that led to higher rates of opioid abuse among orthopaedic surgery patients, including younger age, male sex, public insurance, previous opioid use, alcohol abuse, non-opioid drug abuse, tobacco use, depression, and anxiety [19]. 

Although the detrimental effects of perioperative opioid use are well established, the effects of benzodiazepine use in the perioperative period are unclear and have been seldom studied. The present study found that benzodiazepine users were significantly more likely to fill additional opioid prescriptions and experience prolonged opioid use following hand and upper extremity surgery compared to non-benzodiazepine users. A prospective study with a population of hand and upper extremity patients similar to the present study found that benzodiazepine users filled more opioid prescriptions within six months postoperatively compared to patients without a history of benzodiazepine exposure (2.2 opioid refills vs 1.2; p<.001) [21]. In total joint arthroplasty literature, numerous studies have identified benzodiazepine use as a significant predictor of prolonged postoperative opioid use [11,22]. Following total knee and hip arthroplasty, one study reported that benzodiazepine users filled 81% more opioids within three months postoperatively compared to non-benzodiazepine users [11]. Kim et al. observed that benzodiazepine use placed total joint arthroplasty patients at 1.5 times greater odds of developing persistent opioid use within one year postoperatively (adjusted odds-ratio {aOR} 1.42, 95% CI 1.29-1.56) [22].

One study analyzed risk factors and rates of prolonged opioid use in patients undergoing a variety of orthopaedic procedures, including total knee arthroplasty, rotator cuff repair, anterior cruciate ligament (ACL) reconstruction, distal radius fracture open reduction and internal fixation, ankle fracture open reduction and internal fixation, and lumbar discectomy [23]. It found that 28.9% of benzodiazepine users, 59.6% of opioid users, and 69.1% of opioid plus benzodiazepine users were still using opioids after day 30 postoperatively (p<.001). These findings indicate a potentially additive effect when utilizing both opioids and benzodiazepines, and our study identified a similar trend. In our cohort, we observed that 23.1% of benzodiazepine users, 29.1% of opioid users, and 45.0% of benzodiazepine plus opioid users experienced prolonged postoperative opioid use. It is well established that benzodiazepines and opioids modulate each other’s pharmacologic effects, but the effects of benzodiazepine use on postoperative patients remain poorly understood and should be a focus of future research. 

This study has several limitations, including its retrospective nature. This study included three common hand and upper extremity surgical procedures and patients from three institutions in order to increase the generalizability of study findings, however, it is possible that postoperative pain experience may differ between the three procedures analyzed. Although the PDMP system has been validated and proven to be accurate in controlled substance reporting [13], the filling of prescriptions does not necessarily correlate to actual opioid consumption. It is also entirely possible that patients obtained controlled substances illicitly or used old leftover prescriptions during the study period which could have affected refill rates.

## Conclusions

This study found that patients who use benzodiazepines are at a higher risk of filling additional opioid prescriptions and prolonged opioid use following hand and upper extremity surgery. The risk for developing prolonged opioid use appeared to be even higher for patients who used both benzodiazepines and preoperative opioids. Being aware of these effects and identifying the patients at risk, especially by utilizing PDMP databases, is paramount in helping to prevent the adverse outcomes associated with prolonged postoperative opioid use following hand and upper extremity surgery.
